# Porocarcinome sudoral eccrine de la face: tumeur annexielle rare

**DOI:** 10.11604/pamj.2013.14.135.2503

**Published:** 2013-04-07

**Authors:** Mohammed Lakouichmi, Mohamed El Bouihi, Ibtissam Zrara, Saad Lahmiti, Nadia Mansouri Hattab

**Affiliations:** 1 Service de chirurgie Maxillo Faciale Hopital Militaire Avicenne, Marrakech, Maroc; 2 Service d'anatomie pathologie Hopital Militaire Avicenne, Marrakech, Maroc; 3 Service de chirurgie maxillo-faciale et esthétique, CHU Mohammed VI Marrakech, Maroc

**Keywords:** Porocarcinome sudoral eccrine, face

## Abstract

Le porocarcinome sudoral eccrine est une tumeur cutanée maligne à point de départ glande sudorale. Un homme de 48 ans s'est présenté à la consultation avec une lésion simulant un carcinome basocellulaire de la tempe droite. La biopsie de cette lésion a révélé un porocarcinome eccrine. Le siège facial de cette tumeur est très rare. Elle pose un problème diagnostique et thérapeutique. Nous discutons les différents aspects de cette tumeur avec revue de la littérature.

## Introduction

Le porocarcinome sudoral eccrine (PSE) est une tumeur cutanée maligne qui se développe aux dépend des glandes sudorales eccrines. C'est une tumeur rare et dont la localisation faciale est exceptionnelle [1]. Le PSE peut être primitif ou par dégénérescence d'un porome eccrine [2].

## Patient et observation

**Figure 1:**
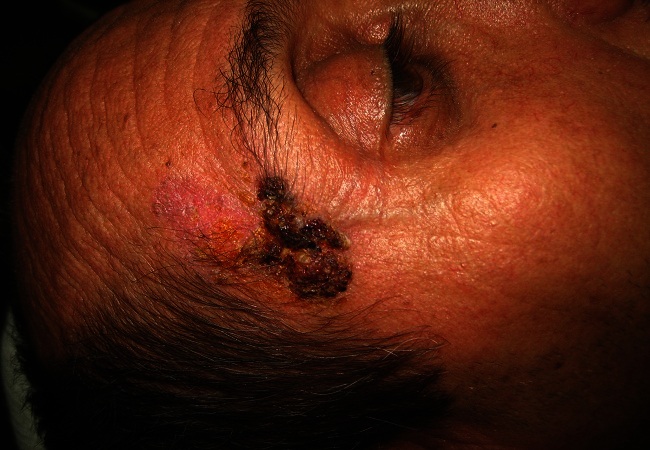
Aspect Clinique verruquo-squameuse simulant un carcinome basocellulaire

**Figure 2:**
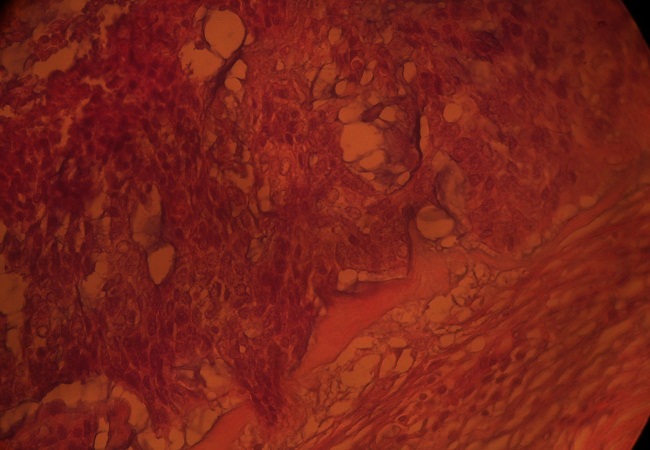
Boyaux kystisés abritant des foyers nécrotiques (HEX40)

**Figure 3:**
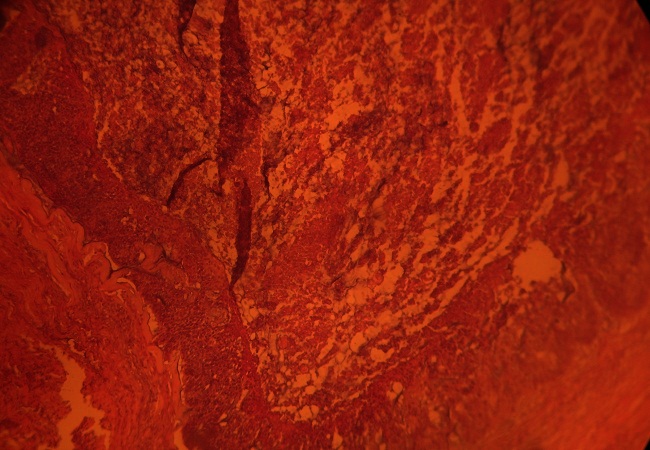
Massifs creusés de lumières glanduliformes (HE X100)

Le patient T R est âgé de 48 ans. Le mois de janvier 2010, il s'était présenté pour une lésion en placard verruquo-squameuse et suintante d'environ 3.5/ 2 cm de la tempe droite (Figure 1). Une biopsie a été réalisée sous anesthésie locale. Le résultat histologique était en faveur d'une prolifération tumorale épithéliale mal limitée, infiltrante et intéressant toute la hauteur du derme. Cette prolifération est composée de boyaux cohésifs creusés par des petites lumières glandiformes conférant un aspect cribriforme ou encore kystisé et abritant des foyers nécrotiques (Figure 2). Les cellules sont peu atypiques mais souvent en mitose. Le cytoplasme ne prend pas le PAS. Le diagnostic d'une tumeur annexielle, le porocarcinome sudoral eccrine, était évoqué. Un bilan clinique, biologique et radiologique d'extension a été réalisé et seulement des petites adénopathies infra-centimétriques de siège parotidienne droit et submandibulaire ont été objectivées. Une exérèse chirurgicale large associée à une parotidectomie exo-faciale droite et un curage ganglionnaire cervical unilatéral droit ont été réalisés. L'étude histologique définitive avec l'immuno marquage avaient confirmés le diagnostic de PSE dont l'exérèse était complète ainsi que l'absence d'envahissement métastatique parotidien et ganglionnaire (Figure 3). Les suites post opératoires ont été simples. Aucun traitement adjuvant n'a été administré au patient et après trois ans aucune récidive n'a été notée.

## Discussion

Le PSE est une tumeur cutanée qui se développe à partir des cellules intra épidermiques et dont le diagnostic est difficile. Un carcinome basocellulaire est souvent évoqué en premier. Il est réputé être très agressif localement avec un potentiel métastatique non négligeable [2]. Il représente environ 0,005% des cancers cutanés [3]. Sa localisation préférentielle est les membres inférieurs avec 50% à 55%, puis la région céphalique pour 20% des cas [4]. Le PSE peut survenir à tout âge entre 19 à 90 ans, mais surtout après l'âge de 60 ans sans prédominance du sexe [5]. Il a été décrit la première fois par Pinkus et Mehregan en 1963 [6]. Cliniquement il se présente sous forme de plaque verruquo-squameuse ou de lésion nodulaire plus ou moins saillante, comme il peut prendre une forme érythémateuse, érosive ou ulcéré avec un diamètre de 1 à 5 cm de grand axe [1]. Parfois la lésion diffuse en surface, sous forme d'une nappe avec des nodules kératosiques sur un fond rouge-jaunâtre et suintant (liée à la composante sécrétoire) [1]. Notre cas clinique illustre cette forme. L'origine des cellules tumorales est intra épidermique au niveau des glandes sudorales eccrines. En effet seule l'étude histologique permet le diagnostic. Elle objective une prolifération épithéliale en îlots avec des foyers de différenciations sudorales, cette prolifération est composée de boyaux cohésifs creusés par des petites lumières glandiformes [4]. L'immuno marquage avec les marqueurs glandulaires (ACE, EMA, a-lactalbumine) permet de confirmer le diagnostic [7]. Le PSE est une tumeur hautement agressive, le potentiel de malignité est essentiellement locale avec un risque majeur de récidive si les marges d'une exérèse large ne sont pas respectées. En outre le risque d'une dissémination métastatique peut être observé dans 20% des cas [2]. Les organes filtres (ganglions, lymphatiques, poumon, foie') sont le siège métastatique habituel. Seule l'atteinte cérébrale semble rare avec un seul cas décrit dans la littérature [8]. Le traitement du PSE au stade localisé est essentiellement chirurgical avec des marges carcinologiques allant de 2 à 3 cm, associées à un curage ganglionnaire adapté au stade évolutif de la tumeur [4]. La chirurgie selon la technique de Mohs est une alternative intéressante en cas de localisation difficile [1]. Les autres moyens thérapeutiques surtout la chimiothérapie et l'immunothérapie, peu efficaces, sont réservées au stade métastatique avec un pronostic sombre [8].

## Conclusion

La marge d'erreur dans le diagnostic du PSE est très importante. L'étude histologique est d'un apport capital dans la prise en charge globale de cette tumeur qui reste exceptionnelle au niveau de la face.
